# Pan-Cancer Analysis of BUB1B/hsa-miR-130a-3p Axis and Identification of Circulating hsa-miR-130a-3p as a Potential Biomarker for Cancer Risk Assessment

**DOI:** 10.1155/2022/3261300

**Published:** 2022-09-22

**Authors:** Xiaoxia Ding, Lele Chen, Danfeng Xu, Yong Yu, Xiaohua Tao, Yibin Fan, Youming Huang

**Affiliations:** ^1^Health Management Center, Department of Dermatology, Zhejiang Provincial People's Hospital, Affiliated People's Hospital, Hangzhou Medical College, Hangzhou, Zhejiang, China; ^2^Department of Dermatology and Venereology, The First Affiliated Hospital of Wenzhou Medical University, Wenzhou, Zhejiang 325000, China

## Abstract

Based on the fact that very little was found in the literature on the question of potential molecules and mechanism for high risk of cancer in patients with psoriasis, this study was designed and performed based on bioinformatics analysis including WGCNA. The most striking result to emerge from the data is that BUB1B/hsa-miR-130a-3p axis, closely related to the development of psoriasis, also plays a remarkable role in multiple cancer development. The expression patterns of hsa-miR-130a-3p were found significantly changed in multiple tumors, which was also associated with prognosis. Additionally, hsa-miR-130a-3p was downregulated in lesion skin of psoriasis, but there was no difference in blood between psoriasis patients and normal controls. Circulating has-miR-130a-3p was found to have a higher level of blood in multiple tumor patients, suggesting that hsa-miR-130a-3p has the potential to be a blood biomarker for cancer risk assessment in patients with psoriasis.

## 1. Introduction

Psoriasis (Ps) is a hyperproliferative chronic inflammatory skin and joint disease with unknown etiology [1], which affects 2-3% of the population. According to previous studies, individuals with psoriasis are at an increased risk of cancer compared to the general population or a reference group without the disease [2, 3], but the underlying association is much less clear [4]. To improve the understanding of the underlying mechanisms of this increased risk, further research is needed.

MicroRNAs (miRNAs) interact with mRNAs and trigger translational suppression or mRNA degradation. MicroRNAs mutation and maladjustment are related to the occurrence and development of human diseases [5]. Accordingly, miRNAs, especially miR-21, miR-125b, miR-146a, and miR-203, may play a role in the pathogenesis of psoriasis. The underlying process affects keratinocytes proliferation and inflammation, as well as T-cell-mediated immunological failure [6]. Notably, plasma miRNAs are also involved in psoriasis pathogenesis targeting the VEGF, MAPK, and WNT signaling pathways [7]. Moreover, changes in miRNA expression or miRNA dysfunction have also been reported to be associated with cancer initiation, progression, and diagnosis in several studies [8, 9]. But still no studies focus on the role of psoriasis-related miRNAs in pan-cancer.

There is growing interest in microarray platforms as a way to detect genetic alterations and to determine biomarkers for many diseases [10]. Several biomarkers and pathways have been implicated in the development of psoriasis and multiple cancer types in previous studies on microarray data. For example, RNF114 was found to correlate with the development of psoriasis and gastric cancer [11]. The findings mentioned above point to a substantial correlation between psoriasis and cancer development. However, there has not been any pan-cancer analysis on the genes involved in the etiology of psoriasis, either mRNAs or miRNAs.

In this study, we analyzed and validated a total of 6 datasets, including blood samples and skin samples of healthy control and patients with psoriasis, by integrated bioinformatics methods. It was found that BUB1B/hsa-miR-130a-3p axis, closely related to the development of psoriasis, also plays a remarkable role in multiple cancer development. This study may provide a new insight into the mechanism of high cancer risk in patients with psoriasis.

## 2. Materials and Methods

**Figure 1:**
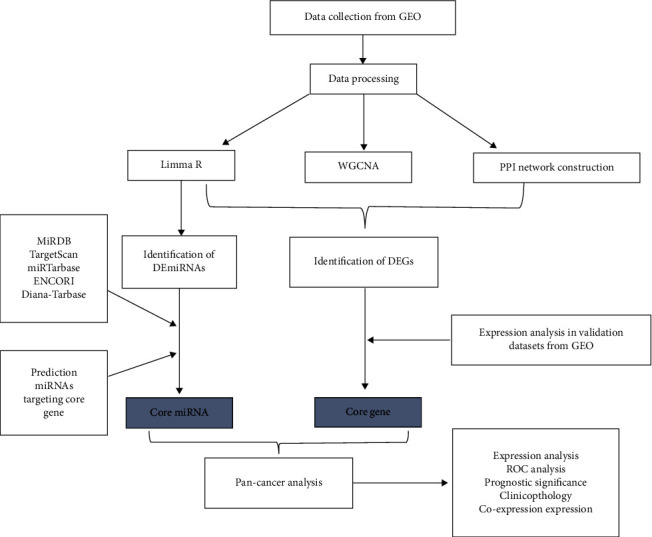
Flow chart.

The flow chart of this study is shown in Figure 1.

### 2.1. Dataset Collection

Gene expression datasets were collected from the Gene Expression Omnibus database (http://www.ncbi.nlm.nih.gov/geo) [12]. The uniformly normalized pan-cancer dataset was downloaded from the UCSC (https://xenabrowser.net/) database [13]: TCGA TARGET GTEx (PANCAN, *N*  = 19131, *G* = 60499). miRNAseq data was obtained from Level 3 BCGSC miRNA Profiling in TCGA (https://portal.gdc.cancer.gov/) ALL (Pan-Cancer) project.

### 2.2. Data Processing and Differential Expression Analysis

The normalized expression matrix of microarray data was downloaded from the GSE dataset. Then, the probes were annotated with the annotation files from the dataset. For merging multiple datasets, we first merged the datasets using the R package inSilicoMerging [14], and then we removed the batch effects [15]. Then, the “limma R” package was used to obtain the differentially expressed genes (DEGs) between the different comparison groups and the control group in the dataset (|Log 2FC| > 1, *p* adj value < 0.05) [16]. Venn plot was drawn between different comparison sets to get overlapped DEGs. Moreover, heatmap and box plot were conducted using “heatmap” and “ggplot2” packages of R software.

### 2.3. Weighted Correlation Network Analysis (WGCNA)

First, we calculated the MAD (Median Absolute Deviation) of each gene separately using the gene expression profile, eliminated the top 50% of genes with the smallest MAD, removed the outlier genes and samples using the goodSamplesGenes method of the R package WGCNA [17], and further constructed a scale-free coexpression network. A power of *β* = 5 was chosen. To further analyze the module, we calculated the dissimilarity of the module eigen genes, chose a cutline for the module dendrogram, and merged modules with distance less than 0.25 along with a sensitivity set to 3. Finally, 14 non-grey modules were obtained. Additionally, we calculated the expression correlation with genes to obtain GS and also calculated the expression correlation of module eigenvectors with genes to obtain MM. Based on the cut-off criteria (|MM| > 0.8 and |GS| > 0.1), 698 genes with high connectivity in the clinical significant module were identified as hub genes.

### 2.4. Functional Correlation Analysis

For gene set functional enrichment analysis, we used GO annotations of genes from the R package org.Hs.eg.db (version 3.1.0) and KEGG annotation from rest API (https://www.kegg.jp/kegg/rest/keggapi.html) as background, mapped the genes to the background set, respectively, and used the R package clusterProfiler (version 3.14.3) for enrichment analysis to obtain gene set enrichment results [18]. Set the minimum gene set to 5 and the maximum gene set to 5000. A false discovery rate (FDR) < 0.05 and *p* < 0.05 were considered significant enrichment.

### 2.5. Construction of the PPI Network

To characterize the crucial DEGs, we used an online tool STRING (https://string-db.org/) to construct PPI networks with a minimum required interaction score of 0.4 [19]. For further analysis, Cytoscape software was used for the download of interaction information. Significant genes were determined by the CytoHubba plugin as hub genes [20]. The significant clusters within the PPI network were selected using the MCODE plugin.

### 2.6. Prediction and Validation of miRNAs Targeting Core Gene

The miRNA target predicting algorithms miRDB (http://mirdb.org/miRDB/) [21], TargetScan [22] (http://www.targetscan.org/), miRTarbase [23] (http://mirtarbase.cuhk.edu.cn/), ENCORI (https://stps://starbase.sysu.edu.cn/) [24], and Diana-Tarbase V8.0 [25] (https://dianalab.e-ce.uth.gr/) were used to predict miRNAs targeting core gene. The intersection of miRNAs obtained from multiple online tools and differently expressed mRNAs (DEmiRNAs) from the GSE142582 dataset was shown in an UpSetR-plot using the UpsetR R package [26].

### 2.7. Expression Analysis of BUB1B and hsa-miR-130a-3p in Pan-Cancer

We extracted the expression data of hsa-miR-130a-3p [MIMAT0000425] from various samples and then performed the log 2 transformation of each expression value. We calculated the difference in expression between paired tumor and adjacent normal tissues as well as normal and tumor samples in indicated tumor types using R software (version 3.6.4) and analyzed the difference in significance using unpaired Wilcoxon Rank Sum Tests. The final results were visualized by ggplot [27]. Moreover, the pan-cancer expression of BUB1B at the protein level was investigated using the Human Protein Atlas database (http://www.proteinatlas.org/) [28].

### 2.8. CancerMIRNome

CancerMIRNome is a comprehensive database with the human miRNome profiles of 33 cancer types from The Cancer Genome Atlas (TCGA) and 40 public cancer circulating miRNome profiling datasets from NCBI Gene Expression Omnibus (GEO) and ArrayExpress [29]. It was used to perform a different analysis of hsa-miR-130a-3p in pan-cancer.

## 3. Results

### 3.1. Data Preprocessing

After searching in the Gene Expression Omnibus database with inclusion criteria including (1) patients with psoriasis and (2) blood samples or skin samples, 6 datasets were chosen, and the detailed information and function are shown in Table 1. Briefly, GSE13355 and GSE14905 were merged to identify differentially expressed mRNAs; GSE142582 was used to explore differentially expressed miRNAs; GSE78097 and GSE55201 were validation datasets for DEmRNAs; and GSE55515 was another validation dataset for DEmiRNAs.

## 4. Differentially Expressed mRNAs in Patients with Psoriasis

**Figure 2:**
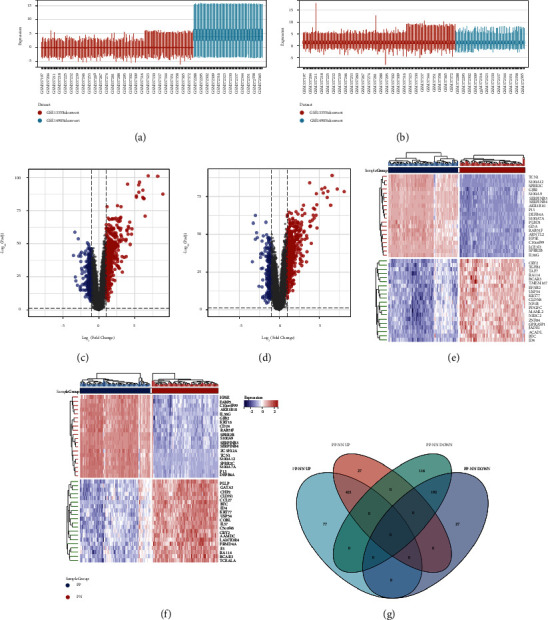
Differentially expressed genes (DEGs) exploring. (a) Box plot before batch effect is removed. (b) Box plot before batch effect is removed. (c) Volcano plot of DEGs from PP-NN set. (d) Volcano plot of DEGs from PP-PN set. (e) Heatmap plot of top 50 DEGs from PP-NN set. (f) Heatmap plot of top 50 DEGs from PP-NN set. (g) Venn plot of overlapped DEGs between 2 sets. (PP: lesion skin from psoriasis patients; PN: no-lesion skin from psoriasis patients; NN: normal skin from normal controls).

Firstly, we removed the batch effects between GSE13355 and GSE14905. From the box plot (Figure 2(a)), we can observe that the sample distribution of each dataset before the batch effect is removed is quite different, suggesting that there is a batch effect, and the data distribution between the various data sets after the batch effect is removed tends to be consistent, and the median is on a line (Figure 2(b)). Then, we identified DEGs from 2 different comparison sets including PP versus NN and PP versus PN. DEGs were identified with the setting of cutoff at FDR < 0.05 and |log 2 (FC)| ≥ 1. The DEGs from the 2 sets were presented as a volcano plot and heatmap plot (Figures 2(c)–2(f)). As shown in Figure 2(g), there are totally 498 upregulated DEGs and 308 downregulated DEGs from PP-NN set, while there are 448 upregulated and 219 downregulated DEGs from PP-PN set. Among them, 421 upregulated DEGs and 192 downregulated DEGs were overlapped between the 2 sets.

### 4.1. WGCNA Analysis and Attainment of Module DEGs

**Figure 3:**
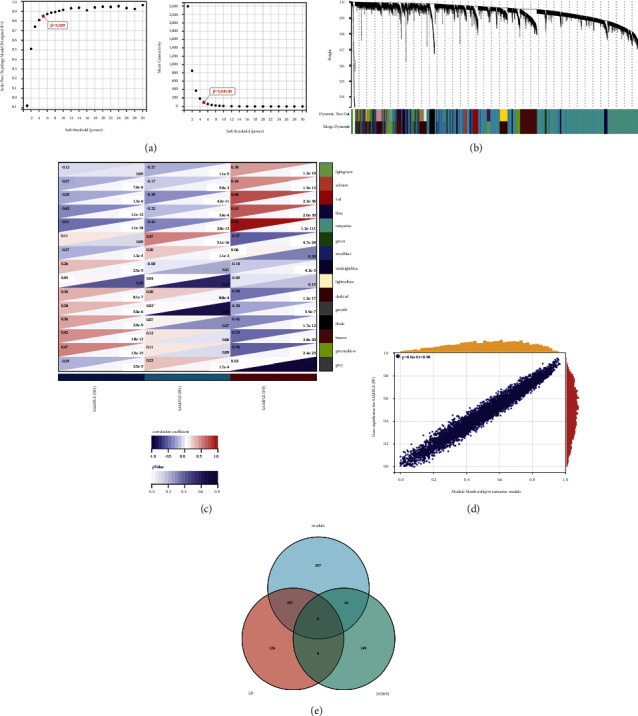
The result of WGCNA. (a) The lowest power for scale independence. (b) Repeated hierarchical clustering tree. (c) The associations between clinic traits and the modules. (d) Scatter plot of GS and MM correlation between turquoise module and PP samples. (e) Venn plot of 697 hub genes from turquoise module and overlapped DEGs from the above 2 different comparisons.

In this study, WGCNA analysis was conducted using the R package WGCNA. The expression patterns of the genes in the same module were similar and relevant to the average linkage clustering. We included 262 samples with clinical traits to filter outlier samples via sample clustering. A soft threshold (*β*) = 5 was set to ensure a scale-free network (Figure 3(a)). Similar modules with a height cut-off value of 0.25 were merged, and 14 non-grey modules were finally obtained (Figure 3(b)). Furthermore, the relationship between the modules and the clinical traits was evaluated to identify the hub module. The results showed that the turquoise module was significantly associated with the PP samples (Figure 3(c)). The module membership and gene significance of turquoise are shown in Figure 3(d). Additionally, we calculated the expression correlation with genes to obtain GS and also calculated the expression correlation of module eigenvectors with genes to obtain MM. Based on the cutoff criteria (|MM| > 0.8 and |GS| > 0.1), 697 genes with high connectivity in turquoise module were identified as hub genes. Subsequently, we plotted the Venn diagram between the modular hub genes and the abovementioned DEGs and finally obtained 297 upregulated genes and 43 downregulated genes (Figure 3(e)).

### 4.2. PPI Network Construction and Hub Genes Attainment

**Figure 4:**
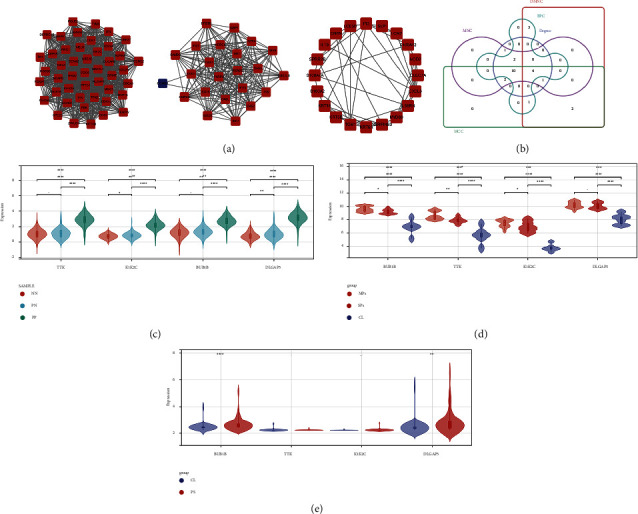
Further analysis of hub DEGs. (a) Clusters from MCODE in Cytoscape. (b) Overlapped genes of 5 different methods from CytoHubba plugin in Cytoscape. (c) Expression pattern of 4 overlapped genes in merged dataset (GSE13355 and GSE14905). (d) Expression pattern of 4 overlapped genes in GSE78097. (e) Expression pattern of 4 overlapped genes in GSE55201.

First, we used the STRING database and Cytoscape software to construct the network of the aforesaid DEGs from the turquoise module, containing 259 nodes and 1804 edges. The top three significant clusters (Figure 4(a)) within the PPI network were selected using MCODE plugin in Cytoscape software (Clusters 1, MCODE score = 42.978; Clusters 2, MCODE score = 19.818; Clusters 3, MCODE score = 5.053). Then, the CytoHubba plugin was used to explore hub genes, and the top twenty were generated using DMNC, MCC, degree, EPC, and MNC methods. The intersection of the top 20 genes obtained from the five calculation methods was presented as an upset plot (Figure 4(b)). A total of 4 overlapped genes were obtained (TTK, KIF2C, BUB1B, and DLGAP5).

### 4.3. Expression Pattern Analysis of 4 Core Genes in the Validation Dataset

To further obtain more core genes, we analyzed the expression patterns of the four genes in the merge dataset as well as the validation dataset, GSE78097 and GSE55201. All 4 genes were highly expressed in psoriatic lesions compared to normal skin from psoriasis patients and normal controls (Figure 4(c)), but no difference was found between normal skin from psoriasis patients and normal controls. As shown in Figure 4(d), BUB1B, TTK, and KIF2C were more highly expressed in psoriatic lesions compared to normal skin, which were more obvious in moderate psoriatic lesions than severe lesions. Differences in the DLGAP5 expression pattern exist only between lesions and normal skin, independent of lesion severity. Additionally, the expression of BUB1B and DLGAP5 in the blood samples of psoriasis patients was also higher than that of normal controls (Figure 4(e)). Combining the validation results of the above two datasets, BUB1B and DLGAP5 were considered to be core genes in the development of psoriasis. DLGAP5 has been reported to be correlated with clinical prognosis, immune cell infiltration, and tumor mutational burden across multiple tumors in previous studies [30]; thus, BUB1B was chosen for further analysis.

### 4.4. Identification and Function Enrichment Analysis of DEmiRNAs of Patients with Psoriasis

**Figure 5:**
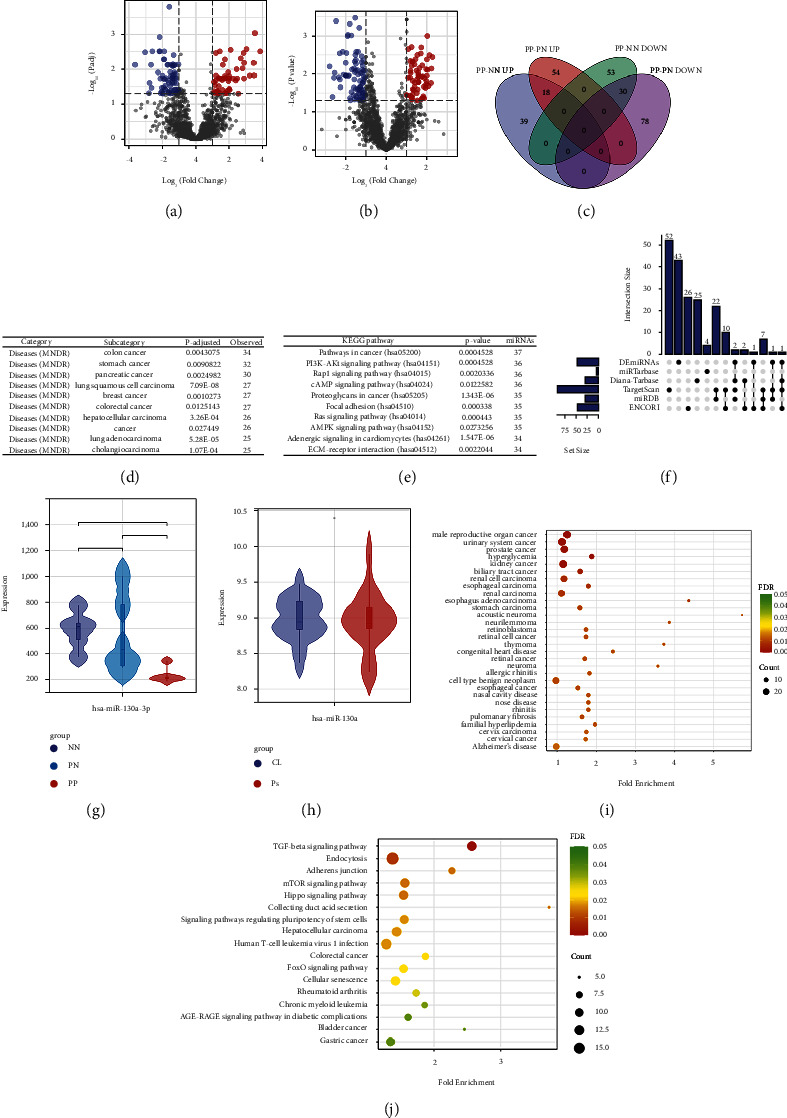
Differentially expressed miRNAs (DEmiRNAs) exploring and analysis. (a) Volcano plot of DEmiRNAs from PP-NN set. (b) Volcano plot of DEmiRNAs from PP-PN set. (c) Venn plot of DEmiRNAs from the above 2 comparisons. (d) Results of disease enrichment analysis of overlapped DEmiRNAs from 2 sets using miEAA. (e) Results of KEGG enrichment analysis of overlapped DEmiRNAs from 2 sets using mirPath. (f) Upset plot of overlapped DEmiRNAs and predicted miRNAs targeting BUB1B using different online tools. (g and h) Expression pattern of hsa-miR-130a-3p in GSE142582 (g) and GSE55515 (h). Results of diseases enrichment analysis (i) and KEGG enrichment analysis (j) of hsa-miR-130a-3p using CancerMIRNome.

Differently expressed miRNAs (DEmiRNAs) of the GSE142582 dataset were obtained from 2 comparison sets including PP-PN and PP-NN. As shown in Figures 5(a)–5(c), a total of 140 DEmiRNAs for PP-NN set (57 upregulated and 83 downregulated) and 180 DEmiRNAs from PP-PN set (72 upregulated and 108 downregulated) were gained. Among them, 48 DEmiRNAs were overlapped between the above 2 sets (18 upregulated and 40 downregulated). Then, all the overlapped DEmiRNAs were used for DO enrichment analysis by miEAA. As shown in Figure 5(d), we ranked the results by the number of engaged DEmiRNAs, and the most involved diseases were multiple types of cancers. Additionally, mirPath (v 3.0) from DIANA-Tools is also used for KEGG enrichment analysis of those DEmiRNAs combined with DEGs from the hub module of the result of WGCNA. As shown in Figure 5(e), the top 1 involved pathway was cancer-related.

### 4.5. Prediction and Validation of miRNA Targeting BUB1B

Firstly, we predicted miRNAs targeting BUB1B using 5 different databases. The intersection of the predicted miRNAs with all overlapped DEmiRNAs is shown in Figure 5(f). With a threshold of simultaneous occurrence in at least three databases, we obtained the only DEmiRNA targeting BUB1B, hsa-mir-130a-P3. Subsequently, we validated the expression pattern of hsa-miR-130a-3p in GSE142582 and GSE55515. As shown in Figure 5(g), hsa-miR-130a-3p was lowly expressed in the PP group compared with both the PN and NN group, while there was no statistical difference between the PN and NN groups. Conversely, the expression pattern of hsa-miR-130a-3p showed no difference in blood samples of psoriasis and normal controls (Figure 5(h)). The functional enrichment analysis was performed through CancerMIRNome. As shown in Figure 5(i), the diseases hsa-miR-130a-3p engaged in were the most cancer-related. The involved KEGG pathways are presented in Figure 5(j).

### 4.6. BUB1B Expression Analysis in Pan-Cancer

**Figure 6:**
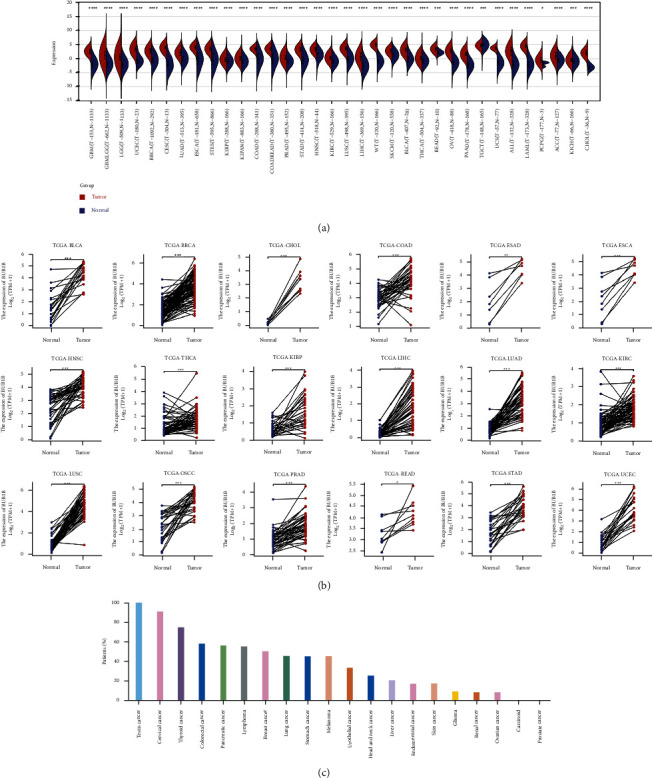
BUB1B expression pattern in pan-cancer; RNA expression pattern between normal and tumor samples (a) as well as paired tumor and normal tissues (b); protein expression pattern in pan-cancer using HPA database.

We calculated the difference in expression between normal and tumor samples in each tumor using R software (version 3.6.4) and difference significance analysis using unpaired Wilcoxon Rank Sum and Signed Rank Tests. As shown in Figure 6(a), we observed significant upregulations in 33 tumors, including GBM, GBMLGG, LGG, UCEC, BRCA, CESC, LUAD, ESCA, STES, KIRP, KIPAN, COAD, COADREAD, PRAD, STAD, HNSC, KIRC, LUSC, LIHC, WT, SKCM, BLCA, THCA, READ, OV, PAAD, UCS, ALL, LAML, PCPG, ACC, KICH, and CHOL, and significant deregulation in THYM. For paired tumor and normal tissues in TCGA pan-cancer (Figure 6(b)), BUB1B was expressed at high levels in 18 tumors, including BLCA, BRCA, CHOL, COAD, ESAD, ESCA, HNSC, THCA, KIRP, LIHC, LUAD, KIRC, LUSC, OSCC, PRAD, READ, STAD, and UCEC. We continue to explore the protein level of BUB1B in pan-cancer using the HPA database. It was found that more than 50% of patients with 10 cancers exhibited high expression, including testis cancer, cervical cancer, thyroid cancer, colorectal cancer, pancreatic cancer, lymphoma, breast cancer, lung cancer, stomach cancer, and melanoma (Figure 6(c)).

### 4.7. Expression Analysis of hsa-miR-130a-3p in Pan-Cancer

**Figure 7:**
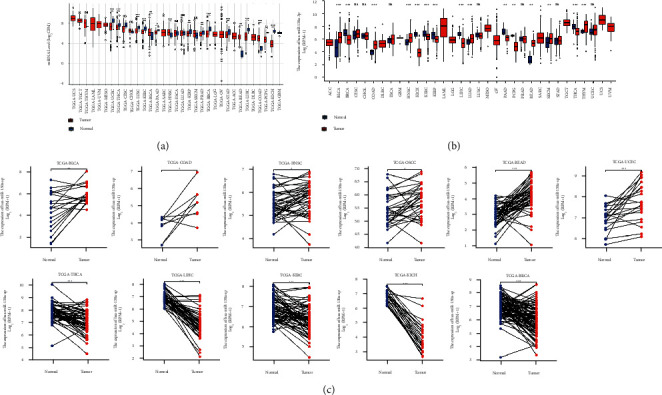
hsa-miR-130a-3p expression pattern in pan-cancer between normal and tumor samples ((a) figure from CancerMIRNome; (b) download dataset from TCGA) as well as paired tumor and normal tissues (c).

After searching “hsa-miR-130a-3p” in “Query” section of CancerMIRNome (http://bioinfo.jialab-ucr.org/CancerMIRNome/), we obtained Figure 7(a). It was observed that hsa-miR-130a-3p had significant upregulations in 4 tumors (BLCA, HNSC, READ, and COAD) and downregulations in 12 tumors (UCEC, THCA, LUSC, KIRC, PAAD, KIRP, PRAD, BRCA, STAD, LIHC, PCPG, and KICH). As for the expression patterns analysis result from download dataset (Figure 7(b)), hsa-miR-130a-3p was found to have significant upregulations in 7 tumors (BLCA, COAD, HNSC, LUAD, READ, SKCM, and THYM) and downregulations in 8 tumors (BRCA, KICH, KIRC, KIRP, LIHC, PAAD, PCPG, and THCA). In paired comparison (Figure 7(c)), hsa-miR-130a-3p had significant upregulations in 6 tumors (BLCA, HNSC, OSCC, READ, COAD, and UCEC) and downregulations in 5 tumors (BRCA, KICH, KIRC, LIHC, and THCA).

### 4.8. Prognostic Significance of hsa-miR-130a-3p in Pan-Cancer

**Figure 8:**
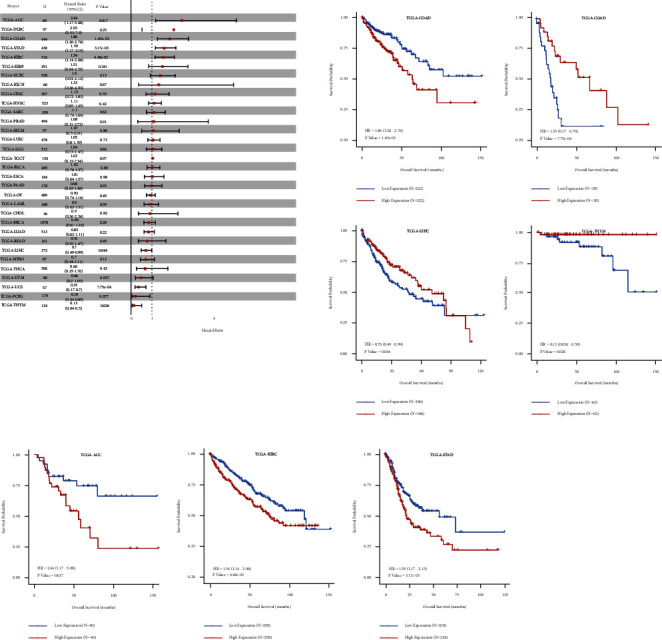
Association between the hsa-miR-130a-3p expression and OS in cancer patients. (a) A forest plot of hazard ratios of hsa-miR-130a-3p in 32 types of tumors. (b) Kaplan-Meier survival curves of OS for patients stratified by the different expressions of hsa-miR-130a-3p.

Using the CancerMIRNome database's “TCGA pan-cancer section,” cox regression analysis of the results from 33 types of cancer suggested that the hsa-miR-130a-3p expression significantly correlated with OS in 6 types of cancer, including ACC, COAD, STAD, KIRC, LIHC, and UCS. Kaplan–Meier survival curves indicated that the unregulated hsa-miR-130a-3p expression was remarkably associated with poor OS in the ACC, COAD, STAD, and KIRC (Figure 8).

### 4.9. ROC Analysis of hsa-miR-130a-3p in Pan-Cancer

**Figure 9:**
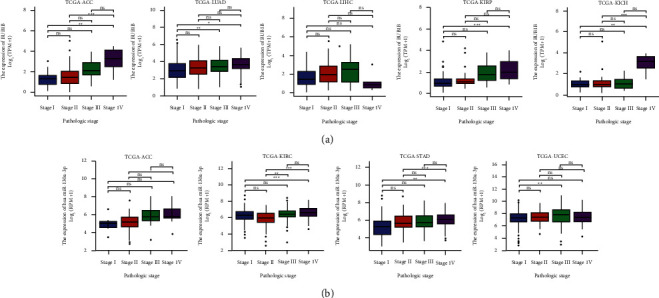
Correlations between the BUB1B and hsa-miR-130a-3p expression and the main pathological stages, including stage I, stage II, stage III, and stage IV.

**Figure 10:**
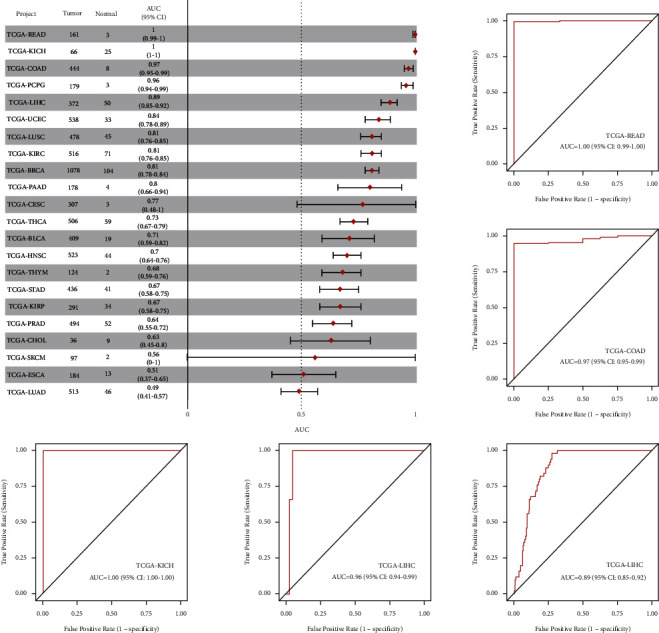
Forest plot and ROC curve of hsa-miR-130a-3p expression in pan-cancer.

The results of ROC analysis of hsa-miR-130a-3p in various tumor types were obtained in the “TCGA pan-cancer part” of CancerMIRNome. As shown in Figure 9, a total of 5 cancers were found to have high AUC value (>0.85), including COAD, KICH, LIHC, PCPG, and READ. The results revealed that hsa-miR-130a-3p expression had excellent diagnostic value in multiple cancer types (Figure 10).

### 4.10. Pan-Cancer Analysis of the Correlation between hsa-miR-130a-3p, BUB1B Expression, and Clinicopathology

The expression of hsa-miR-130a-3p and BUB1B was assessed in cancer patients with different stages (I, II, III, and IV) to discover whether it is associated with clinicopathological features in multiple cancers. The results from the TCGA database revealed that the expression of hsa-miR-130a-3p was significantly upregulated in several advanced cancers, including ACC, KIRC, STAD, and UCEC (Figure 9(b)). As for BUB1B, upregulation in advanced ACC, KICH, KIRC, KIRP, and LUAD was revealed (Figure 9(a)).

### 4.11. Coexpression Analysis for BUB1B/hsa-miR-130a-3p in Pan-Cancer

**Figure 11:**
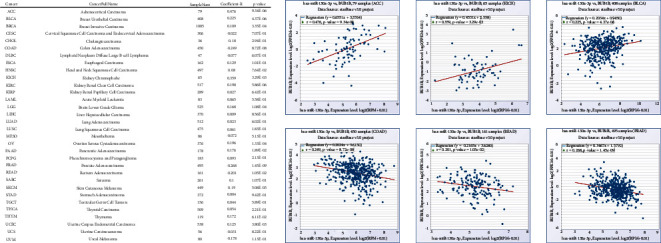
Correlations of coexpression for BUB1B/hsa-miR-130a-3p in pan-cancer.

After searching with hsa-miR-130a-3p and BUB1B in the “pan-cancer” section of ENCORI, we obtained the results shown in Figure 11. It was found that the correlation of BUB1B and hsa-miR-130a-3p coexpression was significant in multiple cancer types, especially in ACC, BLCA, COAD, KICH, PRAD, and READ.

## 5. Discussion

Based on the fact that very little was found in the literature on the question of potential molecules and mechanism for high risk of cancer in patients with psoriasis, this study was designed and performed. The most striking result to emerge from the data is that BUB1B/hsa-miR-130a-3p axis, closely related to the development of psoriasis, also plays a remarkable role in multiple cancer development.

After the identification and validation of DEGs, BUB1B and DLGAP5 were finally found as core genes in the development of psoriasis. In previous studies, several biomarkers as well as pathways have been reported to be correlated with the development of psoriasis, such as apoptosis, cell cycle, angiogenesis, inflammatory response, T cell immune response, VEGF, MAPK, WNT, JAK/STAT, NF-kappa B, and B cell response [7, 31]. It is the first time that BUB1B and DLGAP5 have been linked to the onset of psoriasis, though exact mechanisms are yet unknown. BUB1B, mitotic checkpoint serine/threonine-protein kinase BUB1 beta, is an essential component of the mitotic checkpoint, which is required for normal mitosis progression [32]. An impairment in BUB1B often leads to aneuploidy and chromosome instability, which can contribute to an increased cancer incidence [33, 34]. Furthermore, BUB1B mutations and abnormal expression can contribute to the development of cancer [35]. Consistent with the literature, this research revealed that BUB1B was significantly upregulated in multiple tumors across both paired and unpaired sample analyses, both RNA and protein levels. DLGAP5 (Discs Large Homolog Associated Protein 5), also known as HURP (Hepatoma Up-Regulated Protein) or KIAA0008, was identified as a cell-cycle-regulated protein [36], which is crucial for the movement of the spindle and helps establish the centromere during cell division [37]. According to previous studies, DLAG5 has been shown to be involved in many cancer types, including breast cancer, prostate cancer, and liver cancer [30, 37, 38]. All these results revealed that BUB1B as well as DLGAP5 may bridge the gap between psoriasis and cancers.

In this study, we likewise analyzed differentially expressed microRNAs in skin lesions of patients with psoriasis. KEGG enrichment analysis of these DEmiRNAs showed that they were highly associated with cancer development, which provides further evidence of a potential association between psoriasis and multiple cancers. Among them, hsa-miR-130a-5p was the only miRNA targeting BUB1B. In a series of subsequent analyses on hsa-miR-130a-3p in pan-cancer, we found that hsa-miR-130a-3p expression level was up- or downregulated in a variety of cancer types, some of which was also correlated with the clinical stage. This study supports evidence from previous observations that hsa-miR-130a-3p is a site-specific prognosis biomarker in colorectal cancer [39]. Based on the results of ROC analysis, we also found that hsa-miR-130a-3p was also a specific miRNA for LIHC, KICH, COAD, and PCPG.

Circulating miRNAs appear to be useful for preclinical diagnosis, since they are more sensitive and specific for early diagnosis, risk assessment, and monitoring disease progression [40]. The assays of miRNA in blood samples have been developed as novel, minimally invasive biomarkers for the detection and the risk assessment of cancer [41]. Using the GEO dataset from the CancerMIRNome tool, circulating has-miR-130a-3p was found to have a higher level of blood in multiple tumor patients, which did not differ in blood between psoriasis patients and normal controls, suggesting that circulating has-miR-130a-3p has the potential to be a blood biomarker for cancer risk assessment in patients with psoriasis.

## 6. Conclusion

Through bioinformatics research, we discovered that the BUB1B/hsa-miR-130a-3p axis is closely related to the development of psoriasis as well as several cancer types. Circulating hsa-miR-130a-3p may be a potential biomarker for cancer risk assessment in psoriasis patients. These findings add to the growing body of research linking psoriasis to the development of cancer.

## Figures and Tables

**Table 1:** Detailed information of involved datasets.

Accession	Platform	Disease state	Tissue	Organism	Dataset contents	Purpose	Description
GSE13355	GPL570	Psoriasis and normal	Skin	Homo sapiens	58 PP 58 PN 64 NN	Exploration	PP = involved skin from cases PN = uninvolved skin from cases NN = normal skin from controls

GSE14905	GPL570	Psoriasis and normal	Skin	Homo sapiens	28 PP 28 PN 21 NN	Exploration	

GSE142582	GPL20301	Psoriasis and normal	Skin	Homo sapiens	5 PP 5 PN 5 NN	Exploration

GSE78097	GPL570	Psoriasis and normal	Skin	Homo sapiens	14 MPs 13 SPs 6 CL	Validation	MPs = involved skin from mild cases SPs = involved skin from severe cases CL = normal skin

GSE55201	GPL570	Psoriasis and normal	Blood	Homo sapiens	27 PS 27 CL	Validation	PS = blood from cases CL = blood from controls

GSE55515	GPL11241	Psoriasis and normal	Blood	Homo sapiens	19 PS 15 CL	Validation	PS = blood from cases CL = blood from controls

## Data Availability

The article includes the study's original contributions; further questions should be addressed to the corresponding author.
